# The impact of dietary phosphorus supplementation on postprandial metabolic outcomes: a systematic review

**DOI:** 10.1017/jns.2026.10094

**Published:** 2026-04-17

**Authors:** Rania El Khoury, Omar A. Obeid, Simon Welham, Amanda Avery

**Affiliations:** 1 School of Bioscience, University of Nottingham, UK; 2 Department of Nutrition, American University of Beirut, Lebanon; 3 American University of Beirut, Lebanon; 4 Division of Food Nutrition & Dietetics, University of Nottingham, UK

**Keywords:** Glycaemia, lipidemia, metabolism, phosphorus, thermogenesis

## Abstract

Given the central role of phosphorus in key metabolic processes, including glucose phosphorylation, ATP synthesis, insulin signalling, and energy metabolism, dietary phosphorus availability may influence postprandial metabolic responses. This systematic review evaluates the effects of inorganic phosphorus supplementation on diet-induced thermogenesis, postprandial glycaemia, and postprandial lipidemia in healthy adults. A systematic search of PubMed, Google Scholar, Scopus, and the Cochrane Central Register of Controlled Trials (CENTRAL) was conducted. Only experimental intervention studies assessing phosphorus supplementation as the primary exposure and postprandial metabolic outcomes as primary endpoints were included. Eligible participants were healthy adults aged 18–64 years. Secondary outcomes included changes in body weight, energy intake, and satiety. Ten randomised crossover trials met inclusion criteria, comprising a total of 225 participants. Three out of four studies reported a significant positive association between phosphorus supplementation and diet-induced thermogenesis (*P* < 0.05). Evidence regarding the effects of phosphorus on postprandial glycaemia and lipidemia was inconsistent. An inverse association was observed between phosphorus intake and weight gain (*P* < 0.001) and energy intake (*P* < 0.01), alongside a positive association with satiety (*P* < 0.05). While these findings indicate potential metabolic benefits of dietary phosphorus, particularly in relation to thermogenesis and energy regulation, interpretation is tempered by the small number of available studies, modest sample sizes, and methodological heterogeneity. These limitations restrict causal inference and generalizability. Further rigorously designed, adequately powered clinical trials are therefore warranted to substantiate these associations and to clarify the effects of phosphorus on postprandial glycaemic and lipid outcomes.

## Introduction

Over recent decades, the world has been witnessing gradual but significant changes in eating behaviours, with nutritional transitions from traditional diets rich in complex carbohydrates to diets high in refined sugars, simple carbohydrates, and electrolyte-free commodities.^(1,2)^ Concurrently, there have been considerable global increases in physical inactivity and sedentary behaviour patterns, linked to the rapid advances in technology, urbanisation, and the digital era, thus leading to substantial reductions in overall energy expenditure levels.^(2,3)^ Extensive research has indicated that these overall lifestyle changes have been associated with global increases in the prevalence of metabolic disorders including insulin resistance and abdominal obesity, both classified as major public health challenges.^(4–6)^ The increased consumption of refined cereals and electrolyte-free commodities such as, sugars, oils, and sweeteners have resulted in substantial reductions in the intake of micronutrients, hence compromising their postprandial status.^(2,7)^

Phosphorus is a vital micronutrient that plays an indispensable role in energy metabolism, as it is notably involved in glycolysis, gluconeogenesis, phosphorylation, and cellular insulin signalling. It is responsible for the phosphorylation of glucose to glucose-6-phosphate, an essential step that is required for the cellular glucose uptake and intracellular glucose trapping, necessary for energy production.^(8)^ Apart from this role, phosphorus is also required for the synthesis of adenosine triphosphate (ATP), the energy currency of all cells.^(9)^ These metabolic reactions are all highly dependent on the body’s extracellular phosphorus availability, which is in turn dependent on dietary phosphorus availability.^(10,11)^ Hence, on the metabolic level, any compromises in postprandial phosphorus levels can delay postprandial cellular uptake of glucose, hinder energy production,^(12)^ and eventually increase the risk of developing glucose intolerance and obesity.^(13)^ It is therefore reasonable to deduce that preserving or boosting the status of phosphorus in food, within tolerable limits, would ameliorate such metabolic deviations.

Current UK guidelines recommend a daily phosphorus intake of 550 mg for healthy adults,^(14)^ while US guidelines recommend 700 mg per d.^(15)^ A tolerable upper intake level of 4,000 mg has been established for total phosphorus intake from both food and supplements.^(15)^ To date, adverse effects directly attributable to high dietary phosphorus intake have not been consistently demonstrated in healthy human populations.^(15,16)^ Nevertheless, emerging epidemiological evidence suggests that high intakes of inorganic phosphate salts, particularly from processed foods and additives, may be associated with an increased risk of renal and cardiovascular outcomes, even among individuals without diagnosed kidney disease.^(17,18)^ Importantly, disturbances in phosphate homeostasis, such as hyperphosphatemia, are most commonly reported in the context of non-dietary factors, including impaired renal function or vitamin D toxicity, rather than habitual dietary intake alone.^(15)^

Phosphorus intake is abundant in modern diets due to the widespread use of inorganic phosphorus in food additives and supplements. Nevertheless, it is important to distinguish between inorganic phosphorus, which is highly bioavailable and rapidly absorbed, and naturally occurring organic phosphorus present in dairy products, meat, and other whole foods.^(19–21)^ Organic phosphorus is generally absorbed more slowly and is subject to tighter physiological regulation, which may result in more stable serum phosphate concentrations and potentially different metabolic effects.^(21,22)^ These distinctions indicate that both the chemical form and dietary source of phosphorus may influence postprandial metabolic responses.

Accurately assessing dietary phosphorus intake remains challenging, as the phosphorus content of many foods varies considerably and is often difficult to quantify, particularly in free-living populations.^(23)^ This variability adds uncertainty to observational assessments and complicates comparisons across studies, thereby supporting the use of controlled supplementation protocols to more precisely examine the metabolic effects of phosphorus under standardised conditions.

Beyond source- and intake-related considerations, phosphorus metabolism is tightly regulated by homeostatic mechanisms governing its absorption, distribution, and excretion. Intestinal phosphate absorption occurs via both passive paracellular diffusion and active transcellular transport mediated by sodium-dependent phosphate cotransporters (NaPi-2a, NaPi-2b, NaPi-2c).^(24,25)^ Absorption efficiency is influenced not only by the chemical form of phosphorus and the food matrix, but also by co-ingested nutrients, particularly calcium, which can reduce phosphate uptake through the formation of insoluble complexes.^(19,26,27)^ In addition, differences among inorganic phosphorus salts and variations in the dietary calcium-to-phosphorus ratio may further modulate bioavailability and downstream metabolic responses.^(28,29)^ Once absorbed, circulating phosphate concentrations are maintained within a narrow physiological range through coordinated hormonal regulation involving parathyroid hormone (PTH), calcitriol, and fibroblast growth factor-23 (FGF-23), as well as other modulatory factors such as phosphatonins and blood pH.

Taken together, variability in phosphorus source, bioavailability, and physiological regulation may contribute to the heterogeneous findings reported in studies examining postprandial metabolic outcomes. The aim of the present systematic review was therefore to evaluate the effects of inorganic phosphorus supplementation on diet-induced thermogenesis, postprandial glycaemia, and postprandial lipidemia in healthy adults.

## Methods

### Protocol and guidelines

This systematic review was conducted in accordance with the Preferred Reporting Items for Systematic Reviews and Meta-Analyses (PRISMA-P 2015) guidelines.^(30)^ Its protocol has been registered with the International Prospective Register of Systematic Reviews (PROSPERO*)* with registration ID CRD42023489337.

### Search strategy

This review was designed to answer the following research question: what is the effect of dietary phosphorus supplementation on postprandial metabolism, as compared to the effect of low or depleted phosphorus intake levels, in healthy adults? The PICO (population, intervention, control and outcome) framework for studies’ selection is presented in Table 1. PubMed, Google Scholar, Scopus, and the Cochrane Central Register of Controlled Trials (CENTRAL) were used to search for papers that matched the search strategy. The search strategy was designed to find all human studies that assessed the effect of dietary phosphorus on a wide range of health outcomes. Limiting the search strategy to specific metabolic outcomes was not pertinent, since the initial searches showed very few relevant hits. Therefore, we opted to be less restrictive in the search. A detailed outline of the search strategy used for every database is provided in Supplementary Appendix A. The search process was completed in November 2025.

**Table 1.** Population, intervention, control and outcome (PICO) framework used for study selection

THE PICO FRAMEWORK	
Population	Healthy male and female adults, between the ages of 18 and 64 years.
Intervention	Dietary phosphorus supplementation. Dosage: <4,000 mg of phosphorus per d.
Control	No intervention, placebo, low phosphorus intake, or phosphorus chelators.
Outcomes	Primary outcomes: postprandial metabolism, including thermogenesis (measured by substrate oxidation), postprandial glycaemia (measured by postprandial glucose and insulin levels), and postprandial lipidemia (measured by postprandial lipid levels). Secondary outcomes: body composition, energy intake, and satiety (measured by appetite scores).

### Inclusion criteria

Inclusion criteria was guided by the PICO framework. Only intervention studies with an evaluation of the primary or secondary outcomes described in Table 1 were eligible to be included in this review. Observational studies were excluded. Experimental studies were included if they were randomised controlled trials, parallel or crossover studies, with participants being healthy adults (18 to 64 years), and not having any diagnosed diseases. No language or year of publication limits were applied. No setting restrictions were applied. Studies took place in either community and/or hospital setting.

### Study selection

Initial identified records were merged into one single database and duplicates were removed, using *Zotero Reference Manager*. Relevant abstracts were consequently screened using a title and abstract screening form by a single reviewer. Full text versions were retrieved and independently reviewed by three authors to determine whether inclusion criteria was met.

### Data extraction and data synthesis

A data extraction form was designed and used to recapitulate key details related to each study. We extracted information on the author, study design, study aim, sample size, duration of the study, intervention, measured outcomes, and findings. For the purpose of synthesis, studies were grouped based on the primary outcome assessed. Specifically, the included studies were categorised into three distinct outcome domains: (1) postprandial energy expenditure/diet-induced thermogenesis, (2) postprandial glycaemia, and (3) postprandial lipidemia. This classification facilitated a more structured and meaningful synthesis by enabling comparisons across studies evaluating similar physiological endpoints, despite differences in study design, population characteristics, or intervention type. Thermogenesis was assessed by the mean change in substrate oxidation and resting energy expenditure (kcal/d), glycaemia by the mean change in fasting blood glucose (mg/dl) and insulin levels (μIU/ml), and lipidemia by the percentage change in serum triglyceride, HDL, and LDL cholesterol levels (mg/dl). All studies used the same metrics, allowing for direct comparison without the need for data transformation.

Due to the heterogeneity of studies, in terms of population characteristics (comparisons were made between lean and obese subjects in some studies, while not in others), type of intervention (studies administered phosphorus in different ways: with high protein meals, with carbohydrate meals, or with glucose solutions), and measured outcomes (different metabolic outcomes were measured among the studies), comparing results in a ‘head-to-head’ manner was not applicable, and therefore a meta-analysis could not be conducted. Variation across studies was explored narratively by grouping studies according to their primary outcomes, and by comparing intervention types and population differences to identify potential sources of heterogeneity.

### Quality assessment

The quality of included studies was evaluated according to the Revised Cochrane Risk of Bias Tool for Randomized Trials, ROB2, 2021.^(31)^ Studies were assessed for the following domains: randomisation process, risk of bias arising from period and carryover effects, deviations from intended interventions, missing outcome data, measurement of health outcomes, and selection of the reported result. Each domain was classified as having a high, low, or unclear risk of bias. After pooling together the above assessment domains, an overall bias score was then deduced for each study, along with a classification of it being either a high, unclear, or a low risk-of-bias study.

## Results

### Search results and study characteristics

**Figure 1.**
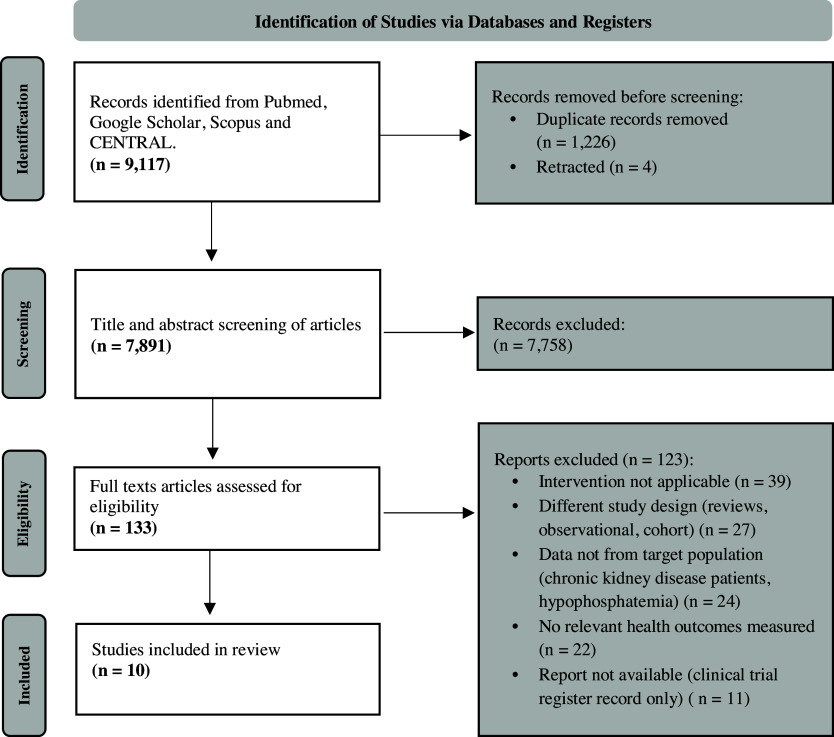
The PRISMA (Preferred reporting items for systematic reviews and meta-analysis) flow diagram of search results.

After removal of duplicates, a total of 7,891 unique abstracts were screened, 133 full-text articles were reviewed and assessed for eligibility, and 10 studies were included, as illustrated in Figure 1. Table 2 presents a summary of the key study characteristics, including study design, population, intervention type, and outcomes assessed. Studies were grouped and presented according to their primary outcome category, and within each category, studies were ordered chronologically. The included studies were all randomised crossover trials (N = 5 single-blinded,^(33,34,36,38,41)^ *N* = 5 double-blinded^(32,35,37,39,40)^ that took place between years 2009 and 2022, and varied between 10 ds to 10 months in duration. Collectively, the studies enrolled a total of 225 healthy participants, 145 males and 80 females, with their baseline body mass index ranging between normal, overweight, and obese.

**Table 2.** Summary of the selected studies used in systematic review

Author, year	Study design	Study aim	N	Sample characteristics	Duration	Intervention	Dosage and form of P supplementation	Measured outcomes	Findings
Bassil et al., 2016 ^(23)^	Double-blind, randomised crossover study	To test the effect of P supplementation on DIT and satiety.	23	10 healthy, lean (normal BMI), male and female adults, and 13 healthy male and female adults living with obesity.	10 d (with 1 week washout period)	Subjects drank a standard 75 g glucose solution (containing no naturally occurring P) with or without P supplements, on 2 separate visits.	500 mg of P, supplemented in the form of potassium phosphate tablets.	REE, RQ, substrate oxidation, appetite scores, and body composition.	P supplementation induced a 23% increase in DIT area under the curve (*P* < 0.05) in subjects with obesity, which was associated with a significant increase in carbohydrate oxidation percentage. Subjects had lower appetite following P supplementation, which was expressed as a significantly lower desire to eat a meal (*P* = 0.02), compared with placebo.
Abdouni et al., 2018 ^(24)^	Single-blind, randomised, crossover study	To investigate the effect of P ingestion on DIT and substrate oxidation.	12	Healthy, lean (normal BMI), male adults.	10 d (with 1 week washout period)	Two groups received isocaloric meals on two separate visits. *Group1*: normal protein (NPr) meal (containing 109 mg of naturally occurring P) administered with or without P. *Group 2*: high protein (HPr) meal (containing 143 mg of naturally occurring P) administered with or without P supplements.	500 mg of P, supplemented in the form of potassium phosphate tablets.	REE, RQ, substrate oxidation, urinary levels of P, creatinine, and urea nitrogen.	NPr and HPr meals had similar DIT that was significantly increased (*P* = 0.005) with P ingestion. A statistically significant difference was detected in DIT according to P and not protein content of the meal.
Assaad et al., 2018 ^(25)^	Single-blind, randomised, crossover study	To determine whether P ingestion with meals can affect postprandial energy metabolism.	15	8 healthy, lean (normal BMI), male adults and 7 healthy male adults living with obesity.	10 d (with 1 week washout period)	A 648 kcal meal composed of white bread, strawberry jam, butter and orange juice (containing 131 mg of naturally-occurring P) was consumed with or without P supplements, on 2 separate visits.	500 mg of P, supplemented in the form of potassium phosphate tablets.	REE, RQ, and substrate oxidation.	P ingestion with meals significantly increased DIT (*P* < 0.01) of both lean and obese subjects and decreased RQ of lean subjects.
El Husseini et al., 2020 ^(26)^	Double-blind, randomised crossover study	To investigate the ergogenic effects of acute P supplementation.	12	Healthy, lean (normal BMI) water polo male players.	2 weeks (with 1 week washout period)	Overnight fasted subjects were asked to cycle for 20 min before ingesting 100 g of glucose with or without P supplements. Three hours later, they were asked to perform a graded cycling exercise for 25 min.	400 mg of P, supplemented in the form of potassium phosphate tablets.	Energy expenditure, RQ, perception of fatigue, exercise efficiency, and heart rate.	P supplementation increased heart rate during exercise performed 3 hours later, but did not affect breathing rate, energy expenditure, RQ, or exercise efficiency.
Obeid et al., 2010 ^(27)^	Single-blind, randomised, crossover study	To investigate the effect of increased P content of a preload solution on subsequent food and energy intake.	53	Healthy, lean (normal BMI), male and female adults.	10 d (with 1 week washout period)	Subjects presented for 2 study d, and were given either water or 50g of sucrose, fructose, or glucose preloads, with or without P supplements. An ad libitum lunch, consisting of standard pizza, was then offered for subjects to consume.	500 mg of P, supplemented in the form of potassium phosphate tablets.	Energy intake (ad libitum) at subsequent meal.	The addition of P to the water preload led to 27% reduction in energy intake at the subsequent meal, similar to that of glucose (25%), while the addition of P to the sucrose or fructose preloads led to 33 and 35% reduction in energy intake at the subsequent meal, respectively.
Shuto et al., 2009 ^(28)^	Double-blind, randomised crossover study	To investigate the acute effect of P supplementation on endothelial function.	11	Healthy, lean (normal BMI), male adults.	Not reported	Subjects were administered a 690 kcal balanced breakfast meal (containing 400 mg of naturally occurring P) with or without P supplements, on two separate visits separated by more than 1 day.	800 mg of P, supplemented in the form of sodium phosphate tablets.	Serum P, blood pressure, FMD, glucose, TG, HDL, LDL, FGF23, PTH, and uric acid.	Serum P level increased significantly 2 hours after the ingestion of a P1200 meal, while did not significantly change after the ingestion of a P400 meal. Serum glucose levels at 2 hours after meal ingestion were not significantly different between both groups. Lipid markers were similar between both groups.
Hazim et al., 2014 ^(29)^	Single-blind, randomised, crossover study	To determine the impact of P ingestion on insulin and postprandial lipidemia.	8	Healthy, lean (normal BMI), male adults.	10 d (with 1 week washout period)	Subjects received a high fat meal (containing 35 mg of naturally occurring P) with or without P supplements, on 2 separate visits.	500 mg of P, supplemented in the form of potassium phosphate tablets.	Serum P, glucose, insulin, TG, NEFA, plasma ApoB100, and ApoB28.	P supplementation increased postprandial serum P levels, but did not affect glucose, insulin, TG, or NEFA levels. P supplementation increased postprandial concentrations of ApoB48 (*P* < 0.05) and decreased that of ApoB100 (*p* < 0.05).
Ayoub et al., 2015 ^(30)^	Double-blind, randomised crossover study	To examine the effects of long term P supplementation on body weight, BMI, WC, and subjective appetite scores.	47	Healthy male and female adults with obesity or overweight.	12 weeks	Placebo or P supplements were ingested with three main meals (breakfast, lunch and dinner) for a period of 12 weeks. These meals were freely selected by the participants based on their usual dietary habits.	375 mg of P with each main meal, supplemented in the form of potassium phosphate tablets, totalling 1,125 mg of P per day.	Body weight, serum phosphate, creatinine, CRP, TC, HDL, LDL, TG, glucose, insulin, and HOMA-IR.	P supplementation significantly reduced WC by 3.62 cm, compared to placebo group (*P* < 0.001). Body weight of the P group was decreased by 0.65 kg, compared to a 1.13 kg increase in the placebo group (*P* = 0.01). Changes in appetite, fullness, and number of snacks consumed were significantly reduced in the P group (*P* < 0.05). Serum P levels, CRP, TC, HDL, LDL, TG, glucose, insulin, and HOMA-IR were similar in both groups.
Volk et al., 2022 ^(31)^	Double-blind, placebo-controlled crossover study	To investigate the acute effect of P supplementation on postprandial levels of serum phosphate, FGF23, postprandial glucose, insulin, and lipids levels.	29	Healthy male and female adults (BMI between 18.5 and 29.9 kg/m^2^).	8 weeks (with 4 to 7 weeks of washout period)	Subjects were administered a 784 kcal balanced test meal (containing 306 mg of naturally occurring P) with or without P supplements.	700 mg of P, supplemented in the form of dihydrogen phosphate tablets.	Serum P, fTRP, fTRCa, PTH, FGF-23, calcitriol, postprandial glucose, insulin, TG, TC, HDL, LDL, and ApoB48 levels.	P supplementation increased serum P levels (*P* < 0.001), compared to placebo group. Postprandial levels of glucose, insulin, and lipids were not substantially affected by P.
Khattab et al., 2015 ^(32)^	Single-blind, randomised, crossover study	To investigate the effect of P ingestion on postprandial glucose and insulin status in healthy subjects.	7	Experiment 1: Healthy, lean (normal BMI), male adults.	10 d (with 3 d washout period)	Subjects attended 3 experimental sessions, that included the consumption of: P supplements Glucose solution (75 g glucose) with P supplements (G + P group). Glucose solution without P supplements.	500 mg of P, supplemented in the form of potassium phosphate tablets.	Serum P, glucose, insulin, insulin sensitivity, and TG.	Ingestion of P alone increased serum P significantly, while ingestion of glucose alone decreased postprandial serum P levels. P supplementation significantly decreased serum glucose and insulin levels (*P* = 0.016 and *P* = 0.002, respectively) at 60 min postprandially.
			8	Experiment 2: Healthy, lean (normal BMI), male adults.	7 d (with 3 d washout period)	Subjects were administered either placebo or P supplements one hour prior to glucose ingestion.			The postprandial increase in glucose levels was similar between the two treatments. Glucose ingestion increased insulin levels of both treatments, but the magnitude of this increase was modestly lower in the P preload treatment group.

P, phosphorus; REE, resting energy expenditure; BMI, body mass index; RQ, respiratory quotient; WC, waist circumference; RMR, resting metabolic rate; CRP, C-reactive protein; TC, total cholesterol; HDL, high density lipoprotein; LDL, low density lipoprotein; TG, triglycerides; HOMA-IR, homeostasis model assessment of insulin resistance; NEFA, non-esterified fatty acids; ApoB100, apolipoprotein B100; ApoB28, apolipoprotein B28.

FMD, flow-mediated dilation; PTH, parathyroid hormone; fTRP, fractional tubular reabsorption of phosphorus; fTRCa, fractional tubular reabsorption of calcium; FGF-23, fibroblast growth factor-23.

### Phosphorus supplementation

**Figure 2.**
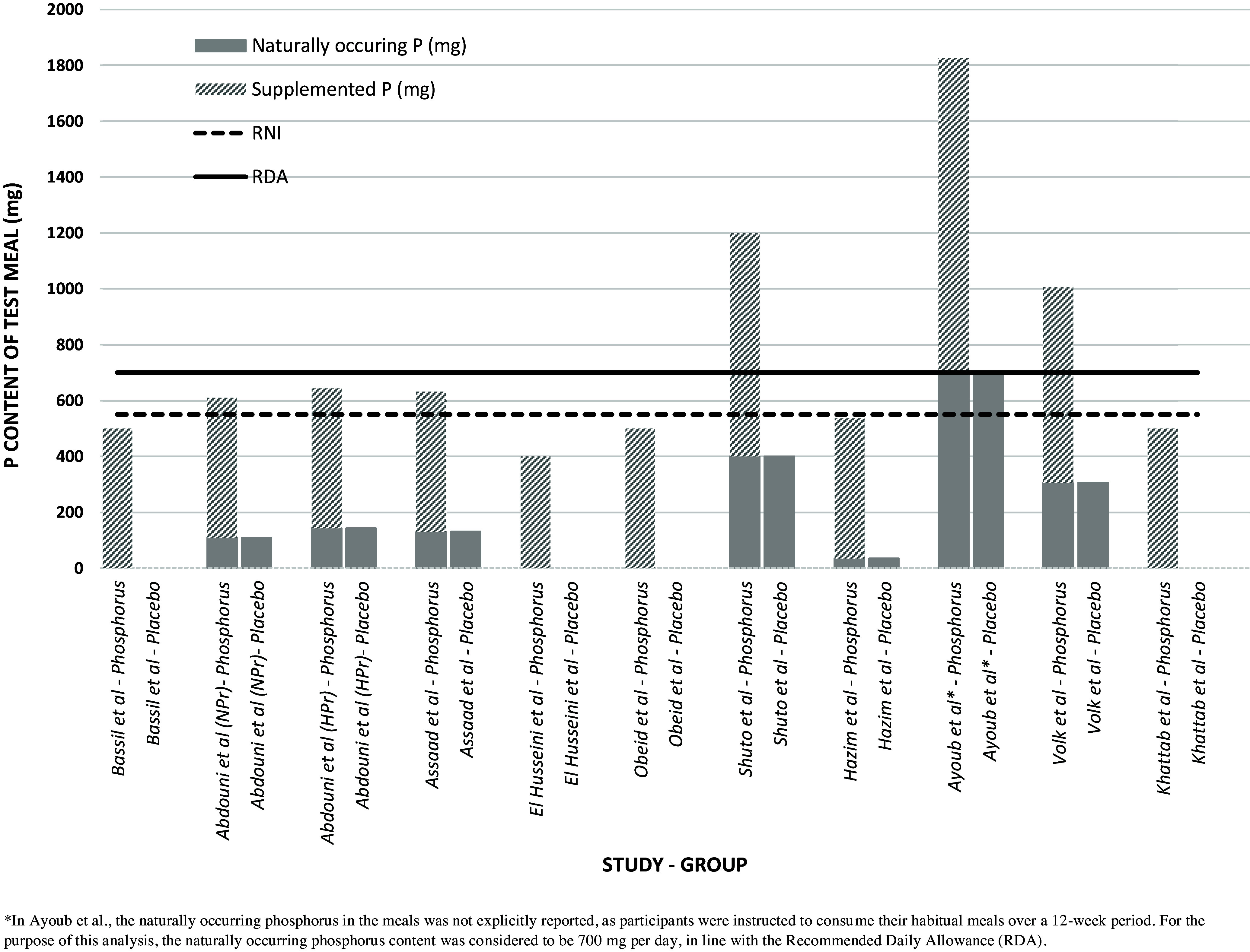
Comparison of dietary phosphorus intake across studies with reference to reference nutrient intake (RNI) and recommended daily allowance (RDA).

Phosphorus supplementation was administered in different forms across the studies: eight studies utilised potassium phosphate, one study used dihydrogen phosphate,^(37)^ and one study employed sodium phosphate.^(40)^ Figure 2 presents a clustered column chart that visually compares the amount of phosphorus supplementation used across studies, relative to the RDA and RNI levels, while distinguishing between supplemental and naturally-occurring sources. Phosphorus supplementation was below the RDA in 7 out of 10 studies. In the remaining three studies — Ayoub et al., Shuto et al., and Volk et al. — the supplementation reached approximately 1,125 mg, 800 mg, and 700 mg per d, respectively. It is important to note that Ayoub et al., specifically investigated the long-term effects of phosphorus supplementation, administering a daily dose of 1,125 mg.^(39)^ If this were considered as acute supplementation, similar to the approach used in the other studies, it would correspond to 375 mg of phosphorus supplemented to each meal. Despite these higher levels of supplementation, all three studies remained well below the upper intake limit of 4,000 mg per d when considering both naturally occurring and supplemented phosphorus.

### Phosphorus and thermogenesis

Three of the four studies investigating the effect of dietary phosphorus supplementation on energy expenditure found a significant positive association between phosphorus exposure and diet induced thermogenesis, compared to low phosphorus intake groups.^(32–34)^ These studies indicated that diet-induced thermogenesis is notably affected and improved by the phosphorus content of meals (Table 2). However, the fourth study reported no effect of phosphorus supplementation on energy expenditure, despite being associated with an increased heart rate during exercise, potentially attributed to a rise in core body temperature.^(35)^ The overall certainty of these findings is moderate, as all four studies showed some concerns in the risk of bias assessment. Despite these concerns, the synthesised findings consistently indicated a positive effect on phosphorus supplementation on thermogenesis. However, the magnitude of this effect varied across studies.

### Phosphorus, body composition, and satiety

Three studies evaluated the impact of dietary phosphorus on satiety, subsequent energy intake, and body weight.^(32,36,39)^ One study found that phosphorus supplementation not only boosted diet-induced thermogenesis in overweight and obese adults, but also significantly lowered their appetite score and improved their postprandial satiety (*P* = 0.02).^(32)^ Similarly, another study revealed that phosphorus content of a preload solution is inversely related to energy intake at a subsequent meal.^(36)^ The third study concluded that although minimal alterations were observed in the postprandial lipidemic and glycaemic parameters, the ingestion of phosphorus was nonetheless able to prevent weight gain and reduce waist circumference by 3.62 cm (*P* < 0.001), as compared with a placebo group, among overweight and obese adults, over just 12 weeks of phosphorus supplementation.^(39)^ Body weight of the phosphorus exposed group was decreased by 0.65 kg, whereas it was increased by 1.13 kg in the placebo group, as compared to baseline values (*P* = 0.01).^(39)^ This study showed that changes in appetite scores, quantity of food to reach fullness, taste of food and number of snacks were significantly reduced in the phosphorus group (*P* < 0.05) as compared with a placebo.^(39)^ Of the three studies, one was classified as high risk and the other two as moderate risk, thereby the overall certainty of the findings is considered moderate.

### Phosphorus and postprandial lipidemia

Four studies examined the effect of dietary phosphorus on postprandial lipidemia.^(37–40)^ Three studies found no association between phosphorus and lipidemia, since no significant changes were seen in postprandial lipid parameters between phosphorus and non-phosphorus exposure groups.^(37,39,40)^ One study found that dietary phosphorus ingestion (500 mg) was able to significantly modify several components of postprandial lipidemia, through the reduction in plasma apolipoprotein B100 (ApoB100) and increase in plasma apolipoprotein B48 (ApoB48).^(38)^ Of the four studies, two were classified as high-risk, one as low-risk, and one as moderate-risk. Given the varying risk of bias across these studies, the overall certainty of the findings is considered low.

### Phosphorus and postprandial glycaemia

In total, five studies tested for the effect of dietary phosphorus on postprandial glycaemia (including studies that tested for glycaemia as a secondary/side objective).^(37–41)^ Four out of five studies found no significant association between phosphorus loading and postprandial glucose and/or insulin levels.^(37–40)^ Conversely, one study found that postprandial glucose and insulin levels were significantly affected by exogenous phosphorus supply, whereby co-ingestion of phosphorus with glucose significantly improved, at 60 min, postprandial glucose (*P* < 0.05), insulin (*P* < 0.05), and insulin sensitivity index (*P* < 0.006).^(41)^ Among the five studies, two were classified as high risk, two as moderate risk, and one as low risk. Given the variation in risk of bias, the overall certainty of the findings is considered low.

### Assessment of risk of bias

Of the ten included studies, one was rated as having low risk of bias,^(40)^ for all five assessment domains (Table 3). Six were rated as having an unclear risk of bias because no explicit information was provided on blinding and randomisation process, nor on the extent of missing data, in addition to some identified concerns regarding the selection of reported results.^(32–35,39,41)^ For instance, one of the studies had few missing reported outcomes in both placebo and phosphorus groups, yet no explanation was given for the reason of this missing data.^(39)^ Three studies were rated as having high risk of bias, owing to the lack of information on participants’ blinding and randomisation process.^(36–38)^

**Table 3.** Risk of bias of included studies, according to the revised Cochrane risk of bias tool for randomised trials

Study	Experimental	Comparator	Outcome	Randomisation process	Bias arising from period and carryover effects	Deviations from intended interventions	Missing outcome data	Measurement of outcomes	Selection of the reported result	Overall
*Basil* et al.^(23)^	Lean adults: glucose solution with and without P	Obese adults: glucose solution with and without P	Energy expenditure; postprandial satiety	Some concerns	Some concerns	Low risk	Some concerns	Low risk	Some concerns	Moderate risk
*Abdouni et al.* ^(24)^	Normal protein meal with and without P	High protein meal with and without P	Energy expenditure	Some concerns	Low risk	Some concerns	Low risk	Low risk	Some concerns	Moderate risk
*Assaad et al.* ^(25)^	Obese adults with and without P	Lean adults with and without P	Energy expenditure	Some concerns	Low risk	Low risk	Some concerns	Low risk	Some concerns	Moderate risk
*El Husseini et al.* ^(26)^	Workload test with glucose load plus P	Workload test without P	Energy expenditure; exercise efficiency	Low risk	Low risk	Low risk	Low risk	Some concerns	Some concerns	Moderate risk
*Obeid* et al.^(27)^	Test meal with P	Test meal with placebo	Energy intake	High risk	Low risk	Low risk	Some concerns	Some concerns	Some concerns	High risk
*Shuto* et al.^(28)^	Test meal with 1200 mg of P	Test meal with 400 mg of P	Endothelial function; glycaemia	High risk	Low risk	Low risk	Low risk	Some concerns	Some concerns	High risk
*Hazim* et al.^(29)^	High fat meal with P	High fat meal with placebo	Lipidemia; glycaemia	High risk	Low risk	Some concerns	Low risk	Low risk	Some concerns	High risk
*Ayoub* et al.^(30)^	Test meal with P	Test meal with placebo	Lipidemia; glycaemia; weight gain; waist circumference	Some concerns	Some concerns	Low risk	Some concerns	Low risk	Some concerns	Moderate risk
*Volk* et al.^(31)^	Test meal with P	Test meal with placebo	Bone metabolism; lipidemia; glycaemia	Low risk	Low risk	Low risk	Low risk	Low risk	Low risk	Low risk
*Khattab et al.* ^(32)^	Glucose solution with P	Glucose solution with placebo	Glycaemia	Some concerns	Low risk	Low risk	Low risk	Some concerns	Some concerns	Moderate risk

P, phosphorus.

## Discussion

The main findings of this review can be summarised as follows: (1) a positive association between phosphorus exposure and diet induced thermogenesis, (2) the impact of phosphorus on postprandial glycaemia and lipidemia remains unclear, (3) an inverse association between phosphorus consumption and weight gain, phosphorus consumption and energy intake, in addition to a positive association between phosphorus consumption and satiety.

Studies included in this review support a potential role of phosphorus in diet-induced thermogenesis. Abdouni et al.,^(33)^ reported that ingestion of 500 mg phosphorus increased postprandial thermogenesis in healthy adults, independent of meal protein content (*P* = 0.005). Similarly, Assaad et al.,^(34)^ observed a modest increase in postprandial energy expenditure of 0.06 kcal/min in both lean and obese participants after 500 mg phosphorus co-ingested with a high-carbohydrate meal (*P* < 0.01). Bassil et al.,^(32)^ demonstrated a 23% increase in postprandial thermogenesis in individuals with obesity following supplementation with 500 mg phosphorus in a glucose solution (*P* < 0.05). In contrast, El Husseini et al.,^(35)^ found no effect on energy expenditure during a workload test, although heart rate increased in the treatment group, potentially reflecting enhanced thermogenic activity. Mechanistically, these effects may be explained by the influence of substrate availability on ATP production. Postprandial increases in glucose and phosphorus enhance cellular uptake of these substrates, facilitating hepatic ATP synthesis.^(10,42,43)^ Phosphorus supplementation may also stimulate insulin secretion, promoting hepatic phosphorus uptake and ATP generation, thereby contributing to diet-induced thermogenesis. Hepatic ATP production has additionally been implicated in satiety signalling via hepatic vagal afferents, which communicate changes in hepatic energy status to the central nervous system.^(44)^ This mechanism may partially account for observations by Bassil et al.,^(32)^ and Obeid et al.,^(36)^ who reported reduced appetite and ad libitum energy intake following 500 mg phosphorus supplementation. Ayoub et al.,^(39)^ further showed that 375 mg phosphorus per meal over 12 weeks reduced appetite, food intake to reach fullness, BMI, waist circumference, and body weight in overweight and obese adults. Although hepatic ATP production provides a plausible mechanistic link to satiety and energy regulation, appetite and energy intake are complex, multifactorial processes influenced by homeostatic, hedonic, cognitive, and environmental factors. Phosphorus-induced modulation of hepatic ATP is therefore unlikely to act in isolation, and observed effects should be interpreted within this broader regulatory context.

Despite the mechanistic plausibility and the observed effects on diet-induced thermogenesis and appetite regulation, the human evidence remains limited. Most studies are small, crossover trials with heterogeneous designs, populations, and phosphorus sources, which restricts generalizability. The small number of studies and modest sample sizes further limit causal inference, and the certainty of the observed associations — particularly regarding appetite, energy intake, and weight regulation — remains moderate to low. Therefore, while preliminary clinical and mechanistic data suggest potential metabolic benefits of dietary phosphorus, these findings should be interpreted with caution, and further well-controlled, adequately powered trials are needed to confirm and clarify these effects.

With regard to postprandial lipidemia, it is hypothesised that phosphorus supplementation would enhance lipid clearance from circulation due to its insulin sensitising capability, given that the synthesis, clearance, and hydrolysis of triglycerides, chylomicrons and lipoproteins are affected by insulin.^(40,41)^ In this review however, no significant changes were seen in postprandial lipid parameters between phosphorus and non-phosphorus exposure groups.^(38–40)^Our findings differ from other comparable studies that were found in the literature (which were not included in this review, due to incompatibility with inclusion criteria). For example, Ditscheid et al.,^(45)^ found that 4 weeks of supplementation with pentacalcium hydroxy-triphosphate (CaP) was able to significantly improve overall cholesterol metabolism, whereby: (1) serum cholesterol concentrations were lower than that of placebo (*P* = 0.008), (2) serum LDL cholesterol and the ratio of LDL: HDL cholesterol were lower after calcium phosphate (CaP) supplementation (*P* = 0.083 and *P* = 0.062, respectively), and (3) bile acid excretion was higher among the CaP group (*P* = 0.003). According to the authors, the above observed beneficial effect of phosphorus supplementation on cholesterol metabolism is not related to an increased clearance of cholesterol, but in reality due to an increased bile acid excretion and a subsequent formation of bile acids from endogenous cholesterol in the liver. This is a consequence of dietary calcium and phosphate precipitating in the small intestine to form insoluble amorphous calcium phosphate (ACP), which binds and inactivates luminal bile acids.^(38)^ Additionally, a pooled review of 21 intervention trials conducted by Trautvetter et al.,^(46)^ found that supplementation with calcium phosphate rather than phosphate only in healthy humans resulted in increased bile acid excretion, decreased blood lipids, and modulation of the intestinal environment, once again through ACP formation in the small intestine. This might explain the results of this review with regards to lipidemia, since the included studies of this review were solely supplemented with phosphorus, overlooking the synergistic role that phosphorus holds when administered with other minerals, especially with calcium.

At the glycaemic level, although phosphorus partakes a crucial role in insulin signalling and carbohydrate metabolism via the phosphorylation of glucose to glucose-6-phosphate, no clear association between phosphorus supplementation and postprandial glycaemia can be deduced from this review. It should be noted, however, that three of the four studies^(37–39)^ that showed no association were not specifically designed to test for the effect of dietary phosphorus on glycaemia. These studies might have failed to detect any association between phosphorus exposure and postprandial glycaemia, since they have measured serum glucose at baseline and/or 120 min post-meal ingestion only, while overlooking the fact that plasma glucose concentrations typically begin to rise at 10 min post meal ingestion, peak at 60 min, and return to pre-prandial levels within 120 to 180 min.^(47)^

Research has increasingly highlighted the role of phosphorus in glucose regulation and energy metabolism in both in vivo and human studies. Animal studies have demonstrated that phosphorus supplementation resulted in lower visceral fat accumulation, higher fat oxidation rates, lower levels of non-esterified fatty acids, lower plasma insulin levels, and improved glucose tolerance, as compared to low phosphorus intake groups.^(48–50)^ Another recently published animal study confirmed that phosphorus supplementation in rats fed a hypercaloric diet, particularly at the 1.5 mg/kcal dose, reduced serum insulin and lipid levels, decreased adipocyte size, and increased energy efficiency.^(51)^ This increase in energy efficiency was attributed to an increased mitochondrial uncoupling resulting from increased uncoupling protein 1 (UCP1) expression, the hallmark protein that stimulates diet-induced thermogenesis in brown adipose tissue.^(51)^ These findings suggest that phosphorus may play a more nuanced role in metabolic health than previously recognised. Similarly, various human studies have shown that serum phosphate levels are negatively correlated with postprandial glucose concentrations and markers of insulin resistance, such as HOMA-IR, and are positively correlated with insulin sensitivity.^(52–55)^ For instance, Haap et al.,^(53)^ found that low serum phosphate levels are significantly associated with reduced insulin sensitivity (*P* = 0.0006) in healthy adults, since no effect on insulin secretion levels was found. Similarly, when white wheat flour was supplemented with phosphorus, magnesium and potassium in a crossover clinical trial, postprandial glucose and triglycerides levels were significantly reduced (*P* = 0.013 and *P* = 0.001, respectively), as compared to the standard white wheat flour group.^(56)^

Although no adverse effects were reported in the intervention studies included in this review, evidence regarding the health effects of higher dietary phosphorus intake remains mixed. Some studies have reported associations between elevated phosphorus consumption — particularly from highly bioavailable inorganic sources — and adverse cardiovascular, renal, and skeletal outcomes.^(17,57)^ Conversely, other investigations have not identified increased health risks associated with higher phosphorus intake in otherwise healthy populations. Under normal physiological conditions, serum phosphate concentrations are tightly regulated; however, excessive intake of inorganic phosphate may disrupt mineral homeostasis, including calcium balance, and could contribute to vascular calcification and elevated cardiovascular risk.^(61–64)^ Importantly, the current evidence remains inconclusive, and the metabolic effects of phosphorus appear to depend on its chemical form, dose, and physiological context.^(65)^ Within this framework, the findings of the present review indicate that phosphorus, when consumed within recommended physiological ranges, may support diet-induced thermogenesis and broader aspects of metabolic homeostasis, suggesting a potential role in optimising rather than compromising metabolic health.

This review may have several potential limitations. First, a limitation at the review-level is that our search was strictly targeted to identify studies that incorporated phosphorus supplementation as their focal and only intervention. As a consequence, we excluded several studies, since many trials tend to test for the effect of phosphorus concomitantly with other minerals like calcium, potassium, and magnesium, given that they are very much physiologically interrelated. In this review, we chose to exclude those studies, because it would be almost impossible to identify the distinctive role of phosphorus, in the case of its supplementation with other minerals. Secondly, at the experimental level, sample sizes of the included studies were often small, somewhat limiting their reliability and generalizability, especially where no power calculations were presented. Furthermore, since all studies were crossover trials and no parallel designs studies were included, we cannot exclude that some carryover effects may be observed between phosphorus exposure and non-exposure groups. One limitation of the synthesis methods used is the variation in study design, population characteristics, and the types of interventions applied across the included studies. This heterogeneity, along with differences in risk of bias, affected the consistency and certainty of the findings, which may limit the generalizability of the conclusions. Finally, the included studies largely did not account for differences between inorganic phosphorus sources or the calcium-to-phosphorus ratio of the background diet, factors that are known to influence phosphorus bioavailability and postprandial metabolic responses. This limitation may contribute to the heterogeneity observed across studies.

Strengths of this review include that it was stringently conducted using the PRISMA guidelines, with double screening of full texts by two independent reviewers, in an attempt to reduce errors and avoid study-selection biases. Another main strength of this overview lies in its search strategy. After mapping out key concepts, we have used a well-defined and controlled set of vocabulary, along with structured synonyms, and title and abstract search terms, all of which paved the way for an efficacious identification of both indexed and non-indexed papers. We are sufficiently confident that had a study been intended and implemented to answer this research question, it would have quite likely been captured in our search. A formal quality assessment of all ten studies was undertaken based on the ‘Revised Cochrane Risk of Bias Tool for Randomized Trial’ guidelines, which further adds to the strengths of this systematic review.

## Conclusion

The available human evidence, although limited and heterogeneous, suggests that dietary phosphorus may support diet-induced thermogenesis and contribute to weight regulation, while no consistent effects have been observed on postprandial glycaemia or lipidemia. These findings indicate a potential role for phosphorus in energy metabolism and metabolic health, but caution is warranted due to the small number of studies, modest sample sizes, and variability in study designs, populations, and phosphorus sources.

Future research should prioritise well-controlled, adequately powered trials to confirm these associations and elucidate underlying physiological mechanisms. Such studies should examine the differential metabolic effects of organic versus inorganic phosphorus, account for absorption efficiency and bioavailability, and consider dietary calcium-to-phosphorus ratios. Comparing phosphorus from whole foods versus processed sources under standardised experimental conditions would enhance ecological validity and translational relevance.

If further confirmed, these findings underscore the potential importance of maintaining optimal phosphorus intake for the primary prevention of metabolic syndrome components in the general population. Dietary recommendations could consider a mineral-to-energy ratio (milligrams of phosphorus per kilocalorie of ingested energy), while ensuring total daily intake remains below the tolerable upper intake level of 4,000 mg. Any consideration of inorganic phosphorus supplementation should be restricted to individuals with normal kidney function, given that those with impaired renal function are contraindicated. Moreover, caution is required, as excessive intake of inorganic phosphate salts has been associated with potential renal and cardiovascular risks.

In summary, while preliminary mechanistic and clinical data suggest beneficial effects of dietary phosphorus on energy metabolism and weight regulation, these conclusions remain provisional. Further rigorous investigations are essential to provide a robust evidence base to guide dietary guidance and potential therapeutic applications.

## Supporting information

10.1017/jns.2026.10094.sm001 El Khoury et al. supplementary material El Khoury et al. supplementary material

## Supplementary material

To view supplementary material for this article, please visit https://doi.org/10.1017/jns.2026.10094
