# Supplementary material for: The impact of dietary phosphorus supplementation on postprandial metabolic outcomes: a systematic review
Source: J Nutr Sci. 2026 Apr 17;15:e26. doi: 10.1017/jns.2026.10094 (PMC13126066; doi:10.1017/jns.2026.10094)
Supplement: El Khoury et al. supplementary material [file S2048679026100949sup001.docx]

**SUPPLEMENTARY APPENDIX A**

**Databases’ Search Strategy:**

| **Database** | **Search strategy** | **Citations** |
| --- | --- | --- |
| ***Cochrane Central Register*** | ***ID Search Hits***   1. dietary phosphorus in Trials 403 2. MeSH descriptor: [Phosphorus] explode all trees 442 3. (phosphorus): TI (title field), AB (abstract field) , KW (keyword field) in Cochrane Reviews, Trials (Word variations have been searched) 1071 | **3,582** |
| ***PubMed*** | ((("phosphorus, dietary"[All Fields] OR "phosphorus, dietary"[MeSH Terms] OR dietary phosphorus[Text Word] OR "phosphorus"[All Fields] OR "phosphorus"[MeSH Terms] OR phosphorus[Text Word] ) AND ( "Clinical Trial" [Publication Type] OR "Clinical Trials as Topic"[Mesh] OR "Controlled Clinical Trial" [Publication Type] OR "Clinical Trial Protocols as Topic"[Mesh] OR "Non-Randomized Controlled Trials as Topic"[Mesh] OR "Pragmatic Clinical Trial" [Publication Type] OR "Clinical Trial, Phase IV" [Publication Type] OR "Adaptive Clinical Trial" [Publication Type] OR "Adaptive Clinical Trials as Topic"[Mesh] OR "Pragmatic Clinical Trials as Topic"[Mesh] OR "Evaluation Study" [Publication Type] )) | **3,461** |
| ***Scopus*** | ALL(fields) (phosphorus* OR dietary phosphorus*) AND (glycemia OR "glucose regulation" OR thermogenesis OR lipidemia OR "postprandial glucose" OR "postprandial response" OR "energy metabolism" OR "glucose metabolism") AND ("clinical trial" OR "randomized controlled trial" OR "cross-over") AND ("postprandial" OR "postprandial metabolism" OR "energy metabolism" OR "metabolism") | **1,094** |
| ***Google Scholar*** | (dietary phosphorus \| phosphorus ) AND (clinical trial \| controlled clinical trial \| placebo \| controlled \| trial \| non-randomized \| randomized \| crossover) | **980** |
